# Take your seats: leftward asymmetry in classroom seating choice

**DOI:** 10.3389/fnhum.2015.00457

**Published:** 2015-08-17

**Authors:** Victoria L. Harms, Lisa J. O. Poon, Austen K. Smith, Lorin J. Elias

**Affiliations:** Department of Psychology, University of SaskatchewanSaskatoon, SK, Canada

**Keywords:** laterality, behavioral asymmetry, hemispheric asymmetry, cognitive processing, motor asymmetry

## Abstract

Despite an overall body symmetry, human behavior is full of examples of asymmetry, from writing or gesturing to kissing and cradling. Prior research has revealed that theatre patrons show a bias towards sitting on the right side of a movie theatre. Two competing theories have attempted to explain this seating asymmetry: one posits that expectation of processing demand drives the bias; the other posits that basic motor asymmetries drive the bias. To test these theories we assessed the real-world classroom seating choices of university students using photographs. A bias for students to choose seats on the left side of the classroom was observed, in contrast to the right side bias observed in theatre seating studies. These results provide evidence in support of a processing-expectation bias.

## Introduction

Despite an overall bilateral symmetry in body morphology, humans display a wide range of motor and perceptual asymmetries (Palmer, 2004; for a review, see Brancucci et al., 2009). Handedness is perhaps the most obvious of these behavioral asymmetries, with approximately 90% of the population displaying a right-hand dominance for writing and the execution of other fine motor tasks (Gilbert and Wysocki, 1992; Dragovic, 2004). Less obvious examples of behavioral asymmetries can also be observed, including biases to turn to the right when entering a room (Scharine and McBeath, 2002), kissing (Barrett et al., 2006), presenting the left cheek when posing for a portrait (Nicholls et al., 1999), or leaning in with the right ear to hear a conversation in a noisy environment (Marzoli and Tommasi, 2009). In addition to these asymmetries, people also exhibit seating asymmetries in movie theatres, airplanes, and classrooms (Farnsworth, 1933; Nicholls et al., 2013; Harms et al., 2014).

Karev (2000) noted a personal observation that seats on the right side of theatres were chosen more frequently than seats on the left. To test this observation, Karev asked participants to select their preferred seating location in a movie theatre from a seating chart of available seats. With the middle seats marked as unavailable, participants were forced to select a seat to the right or left side of the theatre. Consistent with his initial observation, Karev found an overall bias for people to choose seats on the right more often than seats on the left. Although the bias was strongest for right-handed participants, the bias was attenuated, but not reversed, in left- and mixed-handed participants. To explain this asymmetrical seating bias, Karev argued that people, expecting specific processing demands, choose a seating position that will maximize the processing efficiency of the anticipated information content. Seats to the right are preferred in a movie theatre because they position the screen to the left side of the visual field, allowing for efficient processing of the film’s visuospatial and emotional content in the right hemisphere (Bryden, 1982; Corballis et al., 2000).

To test Karev (2000) expectancy hypothesis, Okubo (2010) manipulated both the level of motivation to see the film and the anticipated emotional processing demand of the participants to evaluate their influence on seating behavior using seating charts. A rightward seating bias was observed when right-handed participants were positively motivated to view the film and was absent when right-handed participants were not motivated to view the film. Additionally, when participants were specifically informed that the film contained negative emotional content, the rightward bias was again observed for right-handed participants who were positively motivated to view the film (Okubo, 2010). Okubo argued that these results were consistent with Karev’s (2000) hypothesis that an expectation for emotional processing would bias participants to choose seats to the right.

Using a method similar to Karev (2000), Weyers et al. (2006) examined seating behavior using both the standard seating chart (with the screen positioned at the top of the page) and non-standard seating charts (with the screen positioned either to the far right, the far left, or at the bottom of the page). Consistent with Karev’s (2000) findings, a rightward seating preference was observed for the standard seating chart. This bias was reduced or eliminated when seats were selected from non-standard seating charts; additionally, Weyers et al. (2006) observed a tendency for participants to choose seats to the right side of the paper when selecting a seat in a cinema. Weyers et al. (2006) argued that the observed bias patterns reflect general right-side motor biases and preferences such as a preference for turning to the right upon entering a room.

A common theme across these studies is their reliance on seating-charts. They rely on the assumption that the seat selected when imagining where one would sit in a theatre is equivalent to the seat that would be selected when one actually goes to the movies. Addressing this issue, two real-world studies have examined the seating behavior of actual theatre patrons. Nicholls et al. (2013) assessed the seating preferences of theatre patrons by counting the number of purchased seats for theatre performances booked through Ticketmaster online. Consistent with the seating chart studies, a rightward seating bias was observed for performances at 50% capacity or below. Similarly, Harms et al. (2014) photographed the seating position of movie theatre patrons at actual film screenings. Again, consistent with the seating chart studies, a significant bias for patrons to select seats to the right side of the theatre was observed.

Although much of the seating bias research has focused on a theatre setting, additional studies have examined the influence of seating position in the classroom on academic performance. For example, Farnsworth (1933) noted that academic success was correlated with classroom seating position, with the most successful students seated near the front of the classroom, slightly to the right of center. Similarly, in an examination of spelling performance in children, Morton and Kershner (1987) found that students seated on the right side of the classroom made less spelling errors compared to students seated on the left side of the classroom. An analysis of the types of errors made led the authors to suggest that right-side sitters and left-side sitters employed different processing strategies for completing the spelling test. To further examine the relationship between seating bias and processing strategy, Morton et al. (1993) evaluated how the degree of reliance on specific learning styles varied with seating position in adult participants. Overall, right-sitters were found to rely on responses that showed more artistic or holistic processing and less analytical processing compared to left-sitters. Left- and right-sitters were found to rely on learning styles that emphasized left- and right-hemisphere dominant processes, respectively. For example, left-sitters were found to perform more accurately on dichotic listening tasks using CVC and digit stimuli than right-sitters, reflecting the left hemisphere advantage for verbal processing (Kimura, 1967; Bryden, 1982).

Employing a similar method to Harms et al. (2014), this present study examines the real-world classroom seating preferences of university students. The examination of seating preferences within a classroom setting offers us an opportunity to directly test the competing explanations for seating biases proposed by Karev (2000) and Weyers et al. (2006). Whereas movies are expected to provide predominantly visuospatial and emotional content, university classes are predominantly lecture-based and, thus, are expected to provide predominantly visual and auditory verbal content requiring analytical processing (Ballantyne et al., 1999). Mathematical information (Pinel and Dehaene, 2010), analytical processing (Bever, 1975), and verbal processing (Kimura, 1961) have all been shown to demonstrate left-hemispheric processing advantages. This expectation of left-hemisphere dominant processing demand for university classes gives rise to opposing seating behavior predictions from Karev’s expectancy hypothesis and Weyers et al. (2006) hypothesis that motor asymmetries, such as turning biases, govern seating biases, affording us the opportunity to directly compare their predictions on real-world behavior.

If Karev’s (2000) expectancy hypothesis is accurate, then it follows that an expectation for predominantly verbal and analytical content that is preferentially processed in the left hemisphere should result in a leftward seating bias, as this seating position places the instructor and the projector screen in the right visual field and positions the right ear towards the center of the classroom and the source of the auditory information. This position allows the incoming visual and auditory information to be routed efficiently to the left hemisphere for processing. If the expectation of specific processing demands is what drives seat-choice behavior, then we should see an overall preference for students to choose seats to the left side of the classroom. Alternatively, if Weyers et al. (2006) are correct, and seating biases are more simply a reflection of basic motor biases, then it follows that the difference in expected processing demand between movies and lectures should not influence the seating bias at all. In that case, students in university classrooms should then show the same right side seating bias observed among movie theatre patrons.

## Materials and Methods

### Participants and Procedure

To assess the seating bias observed in classroom seating, the seating position of the students was photographed in lecture halls and classrooms across the University of Saskatchewan campus. Three main classroom clusters where chosen for their high density of classrooms. Over a three-week period for each cluster (for a total of 9 weeks) a research assistant was instructed to enter each classroom before the start of each class period. To ensure that limited availability of seats did not unduly influence seat choice, the number of seated students was counted, and if the number equaled 50% of maximum seating capacity or less, a photograph of the classroom was taken. Classes with greater than 50% of maximum seating capacity were not photographed. A maximum of three attempts were made to photograph each class over the three-week period. To maximize the number of classrooms that met the image selection criteria, all images were taken between five and ten minutes prior to the start of the class.

The images were collected from the center back of the classroom or lecture theatre. The focal length of the lens was adjusted for each image to ensure that all seats in the room were visible in the photograph. A total of 41 images that met the outlined selection criteria were collected from 41 different classes in 29 different classrooms across campus. As this study used the naturalistic observation method, the students in the study were unaware that their seating position was being recorded. This study was conducted following the ethical and procedural guidelines set out by the Ethics Review Board at the University of Saskatchewan.

### Data Coding

A volunteer blind to the hypotheses of the study carried out image coding. The overall seating bias was calculated by counting the number of people seated on the right and left sides of the classroom separately. A laterality index was then calculated by subtracting the number of left-seated students from the number of right-seated students. Thus, a positive score would indicate a right-side seating bias whereas a negative score would indicate a left-side seating bias. Each photograph was then assigned an overall seating bias rating (−1 for left-side bias, 1 for a right-side bias, and 0 for no bias) based on its laterality index score.

In addition to the bias rating, classroom entrance position and location of the center seat were also recorded. Using the floor plan for each classroom or lecture hall where an image was collected, the lateral position (left, right, or bilateral) and axial position (front or back) of the entrance(s) was recorded. The location of the central seat was also recorded and used as the reference point for dividing seating position between the left and right sides of the classroom. Additionally, the subject of each class photographed was recorded and coded as either an Arts or a Science class, according to the class description in the course calendar.

## Results

### Seating Bias

**Figure 1:**
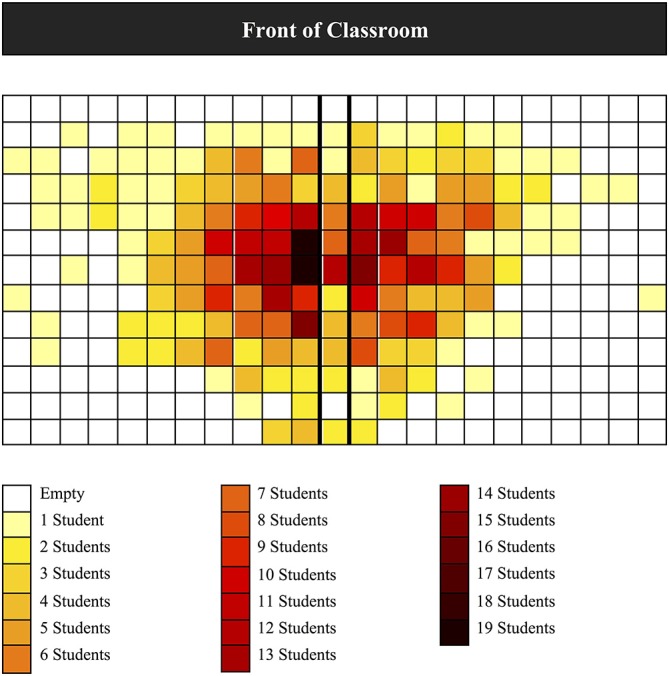
**Normalized seating chart showing the density of students’ seating-choices across all images**. To correct for the differences in seating capacity across rooms, the seating position for each classroom was transposed such that the center seat of the classroom corresponded with the center seat of the seating chart grid.

**Figure 2:**
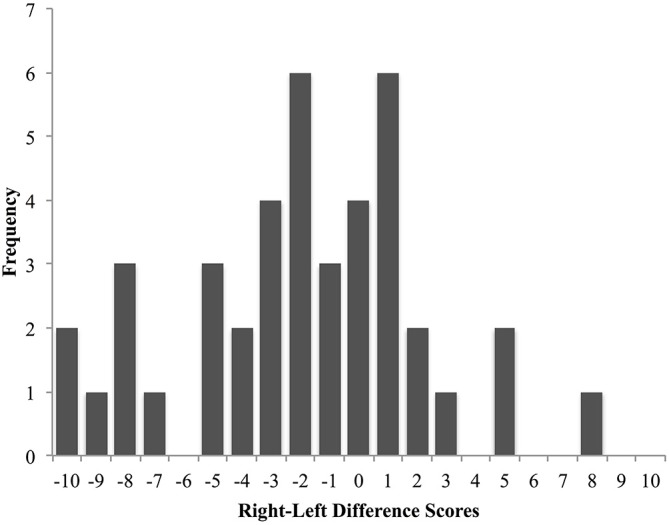
**The frequency distribution of the difference scores calculated by subtracting the number of left-seated patrons from the number of right-seated patrons (right-left) is shown**. Negative values indicate a left-side seating bias. The number of students in the photographs ranged from 1 to 55 with a mean of 17.07 (*SD* = 12.58).

Examining the overall classroom seating bias, a Chi-Square analysis on the frequency of images per seating position category revealed a significant seating bias with left-biased images (*n* = 25; *χ*^2^_(2)_ = 16.439, *p* < 0.001) occurring more frequently than right-biased images (*n* = 12) or no-bias images (*n* = 4). To further assess whether there was a significant difference in the frequency of leftward biased images compared to rightward biased images a second Chi-Square analysis was carried out with the no-bias images removed (*χ*^2^_(1)_ = 4.568, *p* = 0.033). These results indicate a bias for students to select seats to the left side of the classroom. The seating-density pattern observed across all images is presented in Figure 1. Additionally, a one-sample *t*-test was conducted to evaluate whether the overall laterality index across all images revealed a leftward or rightward bias. A significant leftward bias was observed (*t*_(40)_ = −2.999, *p* = 0.005, 95% CI = −3.23, −0.63, *M* = −1.93, *SD* = 4.11). Figure 2 shows the frequency distribution pattern of difference scores across all images.

### Influence of Entrance Location

As the images for the study were collected from a large number of classrooms, it is possible that variations in the location of classroom entrances may have influenced seat choice. Table 1 shows the frequency of left- and right-biased images for each of the three lateral entrance positions. Through *post hoc* analysis, we examined the potential influence of lateral entrance position on seat-choice behavior by testing the interaction of entrance location (right, or bilateral) on seating-bias (left, right). As only one image had a left-side entrance, that image was removed from the analysis to avoid a cell-count violation. The four images showing no bias in seating were also removed for this analysis. A 2 × 2 Chi-Square test was used to assess whether asymmetrical (right-side) or bilateral entrance position influences seating position. No influence of lateral entrance position on classroom seating bias was observed (*χ*^2^_(1)_ = 0.060, *p* = 0.806, *V* = 0.041). These results indicate that any differences in the lateral location of the entrance to the room did not significantly influence students’ choice of seating position.

**Table 1:** **Numbers of left-bias and right-bias images for all lateral entrance positions**.

Entrance position	Left bias	Right bias
Left	1	0
Right	15	8
Bilateral	9	4

Additionally, the location of the entrance with respect to the front or back of the room has also been argued to influence seating position. We examined the potential influence of axial entrance position on seat-choice behavior by testing the interaction of axial position (front, back, or both) on seating bias (left, right). The four images showing no bias in seating were removed for this analysis. A 3 × 2 Chi-Square test demonstrated no influence of axial entrance position on classroom seating bias (*χ^2^*_(2)_ = 0.059, *p* = 0.971, *V* = 0.040; see Table 2). Similar to the results examining lateral entrance position, these results indicate that the presence of a front or rear entrance to the room did not significantly influence students’ choice of seating position.

**Table 2:** **Numbers of left-bias and right-bias images for all axial entrance positions**.

Entrance position	Left bias	Right bias
Front	10	5
Back	10	5
Both	5	2

### Influence of Class Type

Lastly, as processing demands may vary by subject matter, we examined the potential influence of course subject on seat-choice behavior by testing the interaction between class type (Arts, Science) and seating bias (left, right). The four images showing no bias in seating were removed for this analysis. A 2 × 2 Chi-Square test demonstrated no influence of class type on classroom seating bias (*χ^2^*_(1)_ = 0.330, *p* = 0.565, *V* = 0.094; see Table 3).

**Table 3:** **Numbers of left-bias and right-bias images for all class types**.

Class type	Left bias	Right bias
Arts	10	6
Science	15	6

### Comparison of Theatre and Lecture Seating Data

To assess the overall influence of processing demand, we tested the interaction between task type (theatre, lecture) and seating bias (left, right), combining the classroom seating data from the current study with the theatre seating data collected by Harms et al. (2014). Images showing no bias were removed for this analysis. A 2 × 2 Chi-Square test demonstrated a significant influence of task type on seating bias where theatre seating images showed a greater rightward bias and classroom seating images showed a greater leftward bias (*χ^2^*_(1)_ = 11.777, *p* = 0.001, *V* = 0.416; see Table 4).

**Table 4:** **Numbers of left-bias and right-bias images for theatre and lecture task types**.

Task type	Left bias	Right bias
Theatre	8	23
Lecture	25	12

## Discussion

The difference in expected processing demand between movie theatres and university classrooms afforded us a unique opportunity to test two opposing explanations for the rightward seating bias observed in theatre seating studies. Karev’s (2000) expectancy hypothesis predicted that the expectation of left-hemisphere dominant processing demands in the classroom setting would result in an overall leftward preference in seating position among university students, opposite to the rightward bias observed among movie theatre patrons. Alternatively, Weyers et al. (2006) suggestion that rightward motor asymmetries, such as the tendency to turn right upon entering a room (Scharine and McBeath, 2002), dictates seating position preference predicted an overall rightward preference in seating position among university students.

Our naturalistic observation of the classroom seating position of university students revealed a preference for seats on the left side of the classroom, confirming the prediction of a leftward bias derived from Karev’s (2000) expectancy hypothesis. This finding is complementary to Harms et al.’s (2014) observed preference for seats on the right side of the movie theatre using the same procedure as well as the rightward bias observed for online ticket purchases observed by Nicholls et al. (2013). Additionally, a direct comparison of the seating bias for theatre patrons and classroom students provided further evidence that the differing processing demands of the movie theatre and the classroom resulted in opposite seating biases. Together, these studies provide a body of evidence suggesting that lateralized processing asymmetries play a substantial role in governing seating preferences based on anticipated processing demands, with anticipated processing of left-hemisphere dominant verbal and analytical content (Kimura, 1961; Bever, 1975), resulting in a left side seating preference, and right-hemisphere dominant visuospatial and emotional content (Bryden, 1982; Corballis et al., 2000) resulting in a right side seating preference.

It is important to note, however, that hemispheric dominance is not an all-or-none phenomenon. Although individuals may show dominance for left hemisphere language processing, the right hemisphere is far from inactive in processing the linguistic information. It the degree of asymmetry in processing that is thought to play a role in influencing behavior. For example, an individual with a greater degree of left-hemisphere activation compared to right-hemisphere activation during lingusitic processing may be expected to show a stronger, more consistent bias to chose a leftward seat in the classroom compared to an individual with a smaller left-hemisphere advantage. It is overly simplistic to argue that the leftward seating bias observed in the classroom is driven exclusively by the left-hemisphere dominance for linguistic processing, rather, Karev (2000) argues that the seating bias is driven by a relative superiority in left-hemisphere processing at the population level for the given demands of the classroom environment. Additionally, it should be noted that there are individual differences in lateralization of cognitive processing and in real-world behaviors that will influence individual preferences and performance. Where the majority of the population may show left-hemisphere dominance for language processing or a leftward preference for classroom seating, there is always a portion of the population that shows different lateral processing or behavioral biases.

Weyers et al. (2006) found the expected right-side seating preference when the maps of the theatre or cinema were presented in the canonical view, with the screen at the top of the page. When the cinema maps were presented in non-canonical views, the bias shifted to the right side of the page. When presented with both theatre maps and restaurant seating maps, a consistent right side bias was observed relative to the screen and the elevator entrance, respectively. Interestingly, the seating position selected correlated perfectly with the turn at the entrance position, with right-hand turns producing right-side seat choices. This pattern of results lead the authors to argue for a motor asymmetry explanation for the seating bias, such as a right-side turning bias.

Overall, our results are contrary to this prediction. Across all classrooms, students showed a preference for seating positions to the left side of the classroom. Similar to Weyers et al. (2006), the classrooms observed in this study showed a variety of entrance positions. One potential explanation for the leftward seating bias could be the non-canonical positioning of the entrances. The canonical view on a seating chart would place the front of the classroom at the top of the page with the entrance at the rear of the room. Many of the classrooms observed in this study had entrances at the front of the room [corresponding to the bottom position on Weyers et al.’s (2006) charts].

It is possible that these non-canonical entrances required students to make seating decisions based on a view of the classroom where the front of the room was not positioned straight ahead. If this is the case, Weyers et al. (2006) data would predict specific seating distributions for each entrance position: For a front entrance position a right-hand turn would place students to the left side of the classroom. Our data shows a tendency for this leftward bias. However, for a rear entrance to the classroom (the canonical equivalent), a right-hand turn at the entrance would place students to the right side of the classroom. Here, our data shows a tendency in the opposite direction; students are choosing seats to the left side of the room rather than the right. The right and left side entrance positions for the classrooms observed in the present study are not analogous to the rear-entrance, left and right views presented by Weyers et al. (2006) as the entrance position is towards the middle of the left or right side of the classroom. In each of these cases, a right-hand turn at the entrance would either position the student towards the front or the rear of the classroom, rather than the left or the right side.

The leftward bias for classroom seating is also consistent with Morton et al. (1993) finding that preferred seating position reflects preferred learning or response style. For example, they found that left-sitters performed more accurately on CVC and digit dichotic listening tasks compared to right-sitters. They argued that this result reflected a greater reliance on left-hemisphere processing and learning or response strategies in left-sitters. Although the authors continued to suggest that actual seating position likely did not influence performance, other researchers have found visual field and ear advantages for a variety of stimulus processing tasks (e.g., Kimura, 1961; Bryden, 1982; Cherry and Hellige, 1999), suggesting that preferential positioning can improve processing efficiency.

Interestingly, our results do not fit with Farnsworth (1933) and Morton and Kershner (1987) findings of academic performance advantages for students seated on the right side of the room. We did not examine the relationship between seating position and performance, rather we simply examined seating preference in isolation. It may be that left-side seating provides a sense of greater processing fluency without providing a further advantage on a student’s ability to recall or integrate the processed information on assigned tasks; alternatively, right-side seating may provide an attentional advantage that results in greater academic performance, despite a reduced processing fluency for verbal information, that results in improved performance on learning assessments. Additional research is needed to tease apart these possibilities.

We also assessed the potential influence of course differences on student seating position. Although we did not find an influence of general class type (Arts or Science) on seating bias, it may be that the processing demands vary by specific subjects, and that the optimal seating location to improve processing fluency may vary as a function of the topic or subject being covered beyond the general classification of Arts or Science. For example, an Engineering design course and a Fine Arts course may both require a greater reliance on visuospatial (right hemisphere dominant) processing due to the heavy reliance on pictorial information. Similarly, both Mathematics and Linguistics courses rely heavily on symbolic representation processing (left hemisphere dominant). Additional controlled analyses of the relationship between preference, performance, and the differential processing demands per subject or class type are needed to further clarify whether expectation of processing fluency is truly guiding factor in seating location choice among students.

It is worth noting that, besides differences in processing demand expectations, the social contexts surrounding a trip to the movies and attending class are substantively different. Whereas a trip to the movies is typically a social activity with most people attending with friends or family, many students attend classes alone. These contrasting social contexts may be influencing seating behavior. It has been shown that behavior changes based on social context (Berkowitz, 1972), for example, people tend to eat more food when dining with friends compared to dining alone (Hetherington et al., 2006). The social context of the setting may influence how seating location is determined. For example, when selecting a seat with a group of friends, an individual may forgo a preferred seating location to conform to the group preference. This need to conform is negated when an individual is selecting a seating location alone. An examination of seating behavior in theatre and classroom contexts controlling for social factors is needed to assess the relative contributions of both social and processing expectancy factors on seating location asymmetries.

Additionally, personality characteristics such as anxiety level have been proposed as influencing factors in determining an individual’s seating position. An early study by Gur et al. (1976) examined the relationship between seating location and psychopathology in university students. They found that males seated on the right side of the classroom reported higher rates of psychopathology compared to males seated on the left. Conversely, they found that females seated on the left side of the classroom reported higher rates of psychopathology compared to females seated on the right. Similarly, Luck (2006) examined the relationship between seating position choice and psychological distress in patients during a medical consultation. He found a leftward seating bias in patients reporting high levels of anxiety or depression and a rightward bias among patients reporting low levels of anxiety or depression. Given these findings, it is possible that the students who arrived to class and were seated prior to the photographs being taken may be more anxious, and thus more likely to sit to the left side of the classroom. Consistent with this explanation, prior research has shown a pattern between trait anxiety and seating position, with students reporting higher levels of trait anxiety demonstrating a preference to choose seats towards the back of the classroom (Rebeta et al., 1993). An additional examination of the relationship between personality traits and seating position is needed to assess the degree to which anxiety or other personality characteristics might influence seating position.

Although these data provide evidence against the argument that turning biases or rightward motor asymmetries are responsible for the rightward seating biases observed in the theatre-seating studies, it could be argued that the variety of entrance layouts reflected in the data set may have influenced the seating choices of the students. Entrance position, whether lateral or axial, was not found to interact with seating position. The left side seating bias was observed regardless of the position of the room entrance(s). The majority of classrooms had entrances located either solely to the right side of the room (27/41 = 66%) or bilaterally (13/41 = 32%). Only one classroom had a left-side only entrance (2%). Of the 27 right-entrance images, 15 showed a leftward seating bias revealing a greater preference for leftward seats compared to rightward seats at a ratio of 2:1. This suggests that students were willing to cross to the opposite side of the classroom from the right side, where they entered, to take a seat on the left side of the room. This counters the argument that people choose to sit on the side of the room where they enter. Taken together, these observations suggest that entrance position is not a significant factor in determining the location of seat choice within the classroom. However, due to the small data set and the large degree of variability in entrance positions across the images, power is an issue to consider; the data presented here need to be cautiously interpreted in light of this issue. Additional examination of the influence of entrance position and its influence on seating behavior is still needed.

## Conclusion

This naturalistic observation of classroom seating behavior revealed a leftward seating asymmetry, complementary to the rightward asymmetry observed in studies of theatre seating. Consistent with the predictions derived from Karev’s (2000) expectancy hypothesis, the data presented here suggests that lateral biases in seating location are likely not driven by basic motor asymmetries such as turning biases, but rather reflect a behavioral influence of asymmetrical hemispheric lateralization for information processing demands.

## Conflict of Interest Statement

The authors declare that the research was conducted in the absence of any commercial or financial relationships that could be construed as a potential conflict of interest.
